# *In Memoriam* Leon Esterowitz

**DOI:** 10.1117/1.BIOS.3.4.040101

**Published:** 2026-10-29

**Authors:** Vadim Backman, Paul J. Campagnola, Lev T. Perelman, Adam Wax

**Affiliations:** a Northwestern University, McCormick School of Engineering, Evanston, Illinois, United States; b University of Wisconsin – Madison, College of Engineering, Madison, Wisconsin, United States; c Harvard University, Beth Israel Deaconess Medical Center, Center for Advanced Biomedical Imaging and Photonics, Boston, Massachusetts, United States; d Harvard University, Harvard Medical School, Boston, Massachusetts, United States; e Duke University, Department of Biomedical Engineering, Durham, North Carolina, United States

## Abstract

The editorial commemorates Leon Esterowitz, founding director of the NSF Biophotonics program, and his pivotal role in advancing biomedical optics research. It reflects on his scientific contributions, visionary support of emerging research, and enduring impact on the researchers and field he helped shape.

**Figure:**
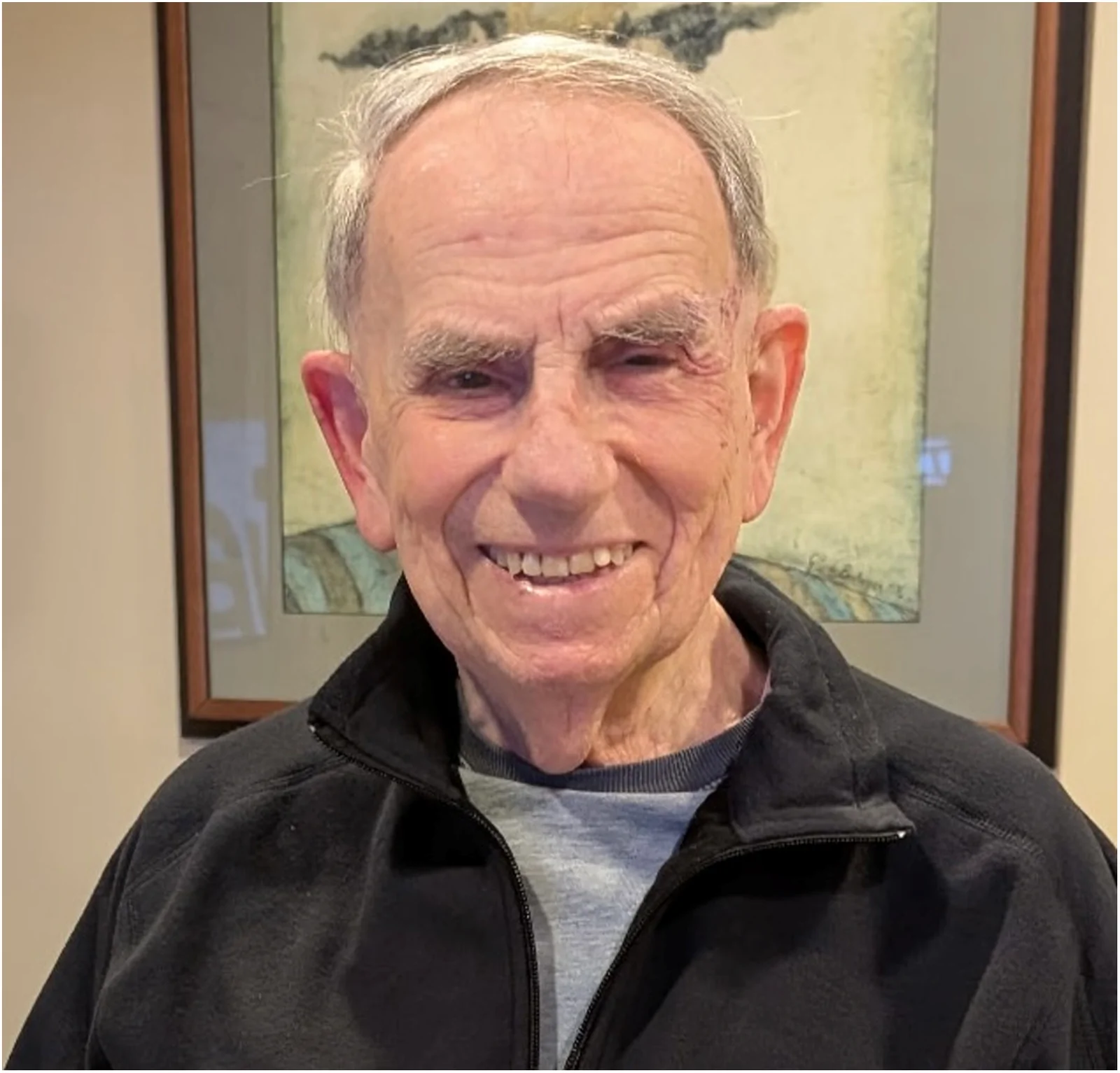
Leon Esterowitz, founding director of the Biophotonics program at the National Science Foundation (photo courtesy of Sohi Rastegar, NSF)

Leon Esterowitz was the founding director of the Biophotonics program at the National Science Foundation (NSF). He passed away peacefully on September 2nd, surrounded by his wife and daughters. His impact on the field was profound, and he will be missed by all the researchers he touched.

Prior to joining the NSF, Esterowitz had a long career at the Naval Research Laboratory, where he served as Section Head, Branch Head and Chief Scientist over a three-decade career. His work focused on next-generation infrared technology, including developing new laser technologies and studying interactions with absorbing media. His research career was impressive, including 332 published papers and 34 patents, including several on medical laser development. His work on advanced solid-state lasers led to new technologies in diode-pumped solid state near infrared lasers that were used in biomedical applications.

In 1999, Esterowitz moved to the NSF to initiate a program devoted to the use of photonics technologies for biomedical applications, bringing the term Biophotonics into common use. He recognized that the rapid expansion of optical telecommunications and enormous investments in laser technologies for defense had produced rapid advances in lasers, optics, imaging, and detection that could be leveraged to develop low-cost, minimally invasive diagnostics but that these had not reached the medical field. Leon was among the first to see the gap between what had become possible in optics and what was actually being used to improve human health. Rather than make a grand declaration about it, he did something characteristically practical and began building a program to address it. What began as a temporary NSF initiative became a permanent biophotonics program. Leon recognized that important new research directions often needed support before they were ready to compete for NIH funding. He saw that NSF could play an important role in identifying and nurturing promising ideas at an earlier stage, giving researchers the opportunity to establish entirely new directions in biomedical optics research. Leon knew both the latest optical technologies and the people working in the field, and he used that understanding to bring new researchers into biophotonics.

Esterowitz clearly loved physics and took genuine pleasure in understanding how things worked. But it also meant a great deal to him to see that knowledge used to help people. His interest in biophotonics was not simply a change in scientific subject matter. It reflected a conviction that knowledge accumulated over a lifetime could and should be directed toward important human problems. In particular, he was passionate about early cancer detection, often relating that members of his own family had succumbed to cancer because there were no early detection methods available. We have all developed early cancer detection methods based on work that Esterowitz not only sponsored through NSF but also helped guide.

The review panels for the Biophotonics program at NSF were not the typical sort. Instead, Esterowitz was interested in spurring new ideas. He would engage the panelists deeply, learning about their work but also seeking to build bridges to the next great advances. He would end each panel with an open-ended discussion of developments in Biophotonics. He mostly listened, but also asked incisive questions. He quickly recognized what was important in the research being discussed, where its real strengths were, and where the problems lay. Often, a single question from Leon was enough to make reviewers reconsider how they were looking at a proposal or uncover the seed of something important that was hidden in an underdeveloped proposal. He did not confuse the most polished proposal with the most important science, and he did not impose his opinions on the panel. He wasn’t there to grandstand or showcase his knowledge, which was considerable, but instead to identify creative new work that could lead to new Biophotonics advances.

Many of us never knew that English was not Leon’s first language. His life story was quite interesting, but, as with so much about himself, he never presented the story dramatically. He was born in France and was still a small child when his family escaped the Nazis. They spent time in Cuba before eventually coming to the United States. He arrived speaking several languages, but not English. Determined to learn it, he tried to finish a book every day and eventually learned English so thoroughly that he entered Yeshiva College as an English major. It was only when he needed to satisfy a science requirement that he chose physics, partly because it seemed to rely less on memorizing facts and discovered the subject that would shape the rest of his life. He earned his PhD in physics from New York University in 1963 and did a postdoc at Yale University. He then led the quantum electronics group at Fort Belvoir, Virginia, before arriving at the Naval Research Laboratory where he spent 28 years of his career.

Instead of retiring, Esterowitz joined the NSF where he served for another 23 years until his retirement in 2022. His legacy at NSF includes over 450 funded awards, representing over $150 million in funding, supporting many of today’s leading technologies in Biophotonics. However, for many researchers, Leon was the sustaining sage who helped them develop vital research programs that continue today.

Leon was a visionary, and yes, he had a beautiful mind. But there was more. Perhaps because as a child he was made to witness the world at its darkest hour, he was a fierce believer in that the goodness of people could make the world a better place. He saw people not by what others saw in them, not by what they had been, but by what they did and what they might become. This is how Leon lived his life. He will be remembered for how many lives he changed.

We were all fortunate to have known him and will miss him.

